# The complete mitogenome of ‘Zaohoucheng’, an important *Saccharina japonica* cultivar in China

**DOI:** 10.1080/23802359.2017.1375877

**Published:** 2017-09-11

**Authors:** Jing Zhang, Nan Li, Na Liu, Tao Liu

**Affiliations:** a School of Biology Engineering, Qilu University of Technology, Jinan, Shandong Province, People’s Republic of China;; b College of Marine Life Sciences, Ocean University of China, Qingdao, Shandong Province, People’s Republic of China

**Keywords:** Cultivar, 'Zaohoucheng', conservative evolution, mitogenome, parental origin, phylogenetic relationship

## Abstract

*Saccharina japonica* is one of the most important economic seaweeds. Here, complete mitogenome of *S. japonica* cultivar ‘Zaohoucheng’ were reported. Its circular mapping organization with the length of 37,657 bp had an overall A + T content of 64.70% and encoded three rRNAs, 25 tRNAs, 35 known mitochondrial proteins and three ORFs. From the total alignment of *S. japonica* and its cultivar ‘Zaohoucheng’, only 12 nucleotide substitutions were detected. Gene arrangement and component of ‘Zaohoucheng’ mitogenome were identical to those of reported *Saccharina* species, indicating highly conservative evolution. The phylogenetic analysis showed that ‘Zaohoucheng’ had a closer relationship with *S. japonica* which strongly supported the parental origin of 'Zaohoucheng'.

*Saccharina japonica* (Laminariales, Phaeophyceae) is one of the most important seaweeds due to its economic value and global distribution (Kain 1979). About 20 cultivars have been bred (Zhang et al. 2016a) in China and ‘Zaohoucheng’ is an important inbred line of *S. japonica* mainly cultured in North China. Here, we determined its complete mitogenome, gave the comparison with that of *S. japonica* and conducted phylogenetic analysis to reveal the genetic and evolutionary characteristics at genomic level.

One ‘Zaohoucheng’ sample (specimen number: 2010084028) was collected from Lidao Bay, Shandong, China (37°13′N, 122°34′E) and stored at −80 °C for DNA extraction. The experimental scheme and data processing were followed by the previous report (Zhang et al. 2011).

Complete mitogenome of ‘Zaohoucheng’ was characterized as a circular molecule with the length of 37,657 bp (GenBank accession no. KX073816). Its mitogenome had an overall AT content of 64.70% exhibiting a high AT richness. Cumulative AT-skew (–0.1218) and GC-skew (0.1660) analysis reflected a light bias toward T and G on H-strand. The mitogenome encoded 66 genes, including three rRNAs (23S, 16S and 5S), 25 tRNAs, 35 protein-encoding genes and three ORFs. With the exception of *rpl*2, *rpl*16, *rps*3, *rps*19, *tat*C and ORF130, 60 genes were encoded on H-strand. The protein-encoding regions were 29,007 bp in length. One conserved gene cluster (*rps*8–*rpl*6–*rps*2–*rps*4) was also found. All protein-encoding genes started with ATG codon and 68.42% terminated with TAA codon, higher than that for TAG (21.05%) and TGA (10.53%).

From the total alignment of *S. japonica* and ‘Zaohoucheng’, 12 nucleotide substitutions were detected which six led to the variety of amino acid. No substitutions were observed in tRNA genes. In conclusion, the component and arrangement of mitogenome were consistent with those of reported *Saccharina* species and cultivars (Yotsukura et al. 2010; Zhang et al. 2011, 2013, 2016b, 2016c), showing highly conservative evolution.

Bayesian analysis based on the whole mitogenome sequences shared by 16 available Laminariaceae algae was utilized to reconstruct the phylogeny. *Ectocarpus siliculosus* as outgroup. Species were divided into two groups: *Saccharina* and *Laminaria* supporting existing taxonomic systems (Yoon et al. 2001; Lane et al. 2006), and all reported cultivars in China were in *Saccharina* clade (Figure 1). Additionally, ‘Zaohoucheng’ first groups with *S. japonica* which validated its parental origin.

**Figure 1.**
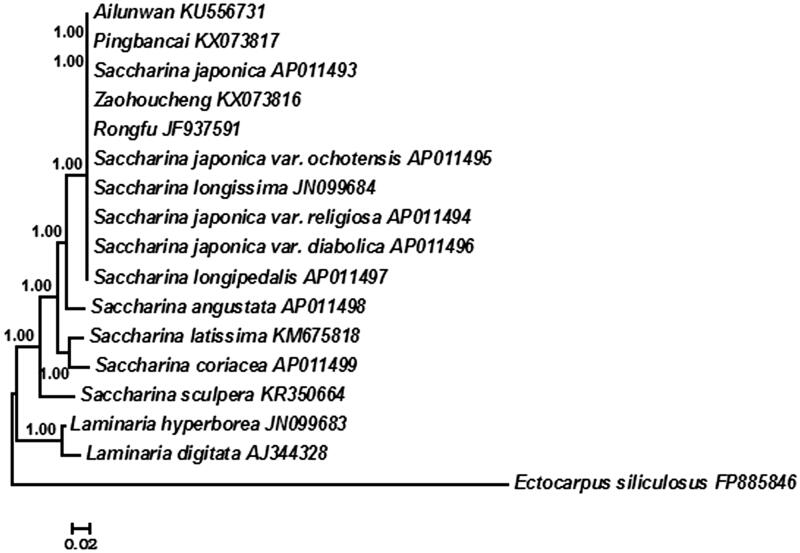
Phylogenetic tree constructed based on combined 35 mtDNA protein-encoding genes using Bayesian analysis.
